# Supplementary material for: Pharmacotherapy of infertility in Ghana: Why do infertile patients discontinue their fertility treatment?
Source: PLoS One. 2022 Oct 17;17(10):e0274635. doi: 10.1371/journal.pone.0274635 (PMC9576038; doi:10.1371/journal.pone.0274635)
Supplement: S1 File — (DOCX) [file pone.0274635.s001.docx]

Supporting Information

Pharmacotherapy of infertility in Ghana: Why do infertile patients discontinue their fertility treatment?

Arhin, M. S.,^1*^, Mensah, K. B ^1^ , Agbeno, E.K..^2^_,_ Diallo, A.A^2^, Henneh, I.T^3^, Ansah, C.,^1^

*^1^Department of Pharmacology, Kwame Nkrumah University of Science and Technology (KNUST), Ghana* || *^2^Department of Obstetrics and Gynecology, School of Medical Sciences, University of Cape Coast, Ghana* || *^3^Department of Pharmacotherapeutics and Pharmacy Practice, School of Pharmacy and Pharmaceutical Sciences, University of Cape Coast, Ghana*

*Corresponding Author

Stephen Mensah Arhin

Department of Pharmacology

College of Health Sciences

Kwame Nkrumah University of Science and Technology, Kumasi, Ghana

Email: [steppahead@gmail.com](mailto:steppahead@gmail.com), [stephen.arhin@ucc.edu.gh](mailto:stephen.arhin@ucc.edu.gh)

[ORCID: 0000-0002-8529-2000](https://orcid.org/0000-0002-8529-2000)

# **Interview transcripts of twenty infertility patients and eight health professionals**

To ensure confidentiality and anonymity, interviewees were coded as Client interviewed Number one (CIN-1), Client Interviewed Number two (CIN-2), up to Client Interviewed Number twenty (CIN-20). Thus, the serial codes begin from CIN-1, CIN-2, CIN-3, CIN-4, and CIN-5… up to CIN-20). Concerning clinical staff, the first interviewee was labeled as Health Practitioner interviewed number one (HP-1), Health practitioner interviewed number two (HP-2), up to Health Practitioner interviewed number eight (HP-8).

**CIN-1**

**Interviewer:** My name is Stephen Mensah Arhin, a PhD student from KNUST, Kumasi. As I introduced myself to you on the first day of your visit to the fertility clinic, I am calling to check up on you as I informed you earlier on. Please we have observed that, you have not been coming for review visit as indicated by the doctor. Because of that, I would like to find out why you sometimes skip/discontinue review appointments with clinicians? This conversation would be recorded for later transcription just for the purpose of this study. Do I have your permission to do that?

**Interviewee:** Okay. No problem with that.

Interviewer: Alright. Thank you. Please may I know why you have not been coming for review as scheduled with the doctor? What really keeps you away?

**Interviewee:** I was not seeing any results and so I resulted to herbal medicine. *“Mebae bae bae na saa na 3te”.* The problem was persisting. The issue is that, it is difficult to wait when you realize you are not seeing results after coming for about five times. When you start with expectation and you do not see the outcome, it becomes worrying. For that reason, I switched to *herbal treatments*. It is not easy at all. Sometimes we are psychologically down and lose confidence. That is why coming for review is sometimes difficult for us. We are only praying that, we will be able to overcome the situation.

**Interviewer:** Did you see any effect after resorting to herbal medications?

**Interviewee:** Now it seems okay, although I am still not pregnant. My stomach used to disturb me a lot, sometimes as if there is air in my stomach….sometimes it make ‘wuuuu’ then it stops, wuuu, then it stops, but now those things are decreasing. I am hoping the pregnancy will follow soon.

**Interviewer:** Alright then. We are hoping to see you at the clinic soon. But before I leave you, is there anything you would like to share with me concerning your experiences at the clinic?

**Interviewee:** well, I don’t have much to say again. Actually, the reception was good. The staff received us well. Although we may sit and wait for some time before doctor comes. Apart from that, there is actually no problem I encountered over there.

**Interviewer:** Thank you very much for having time with me. God bless you. See you soon.

**CIN-2**

Q1. Interviewer: please good afternoon. My name is Stephen Arhin, a PhD student from KNUST, Kumasi. I met you on the first day of your visit to the fertility clinic and discussed with you that we will be checking up on you to find out how the treatment is going and to remind you about review visits. But unfortunately, we have observed that, you missed some of your appointments with the specialist. For that matter, I would like to ask you some few questions as a form of interview to find out why you stopped coming to the clinic. The interview will be recorded on phone just for the purpose of this study. Please I’m I permitted to do so?

**Interviewee:** yes please, you may go ahead.

**Interviewer:** Thank you. Please why you sometimes skip/discontinue review appointments with clinicians?

**Interviewee:** Is my husband oo, he did not want to come and do the test. I told him but still oo daa, does not want to come. I even want you to speak to him for me when he closes from work and we are all sitting together here. He is a driver and sometimes closes at 7pm. He has gone to work at certain place but hopefully he will come today. He was working somewhere but has now been transferred to our place here. When he comes and he is sitting here, then I may call you so that you can speak to him for me, but the problem is that, sometimes he gets home too late. Hoping today he comes early around 7 pm so I can call for you to speak to him for me to see whether he would listen to me. The doctor told me if the man’s results has not come, he would not know how to continue treating me alone. Doctor said he cannot give me medication again, so that day I was not given any medication and I returned home. May be if you are able to talk to him, he may take it serious. It is a serious matter.

Even when I’m coming, he is the one who gives me money, but I still can’t understand why he himself is not willing to come. He even went to a clinic close by to take advice on how to collect semen sample but hmmm.

I get worried because is been almost one year now, if he had come to do the test, I am sure by now God would have glorified himself. As we speak now, even the person who introduced me to that place is having two kids, one of whom is even less than 3 months. Whenever I see the woman, I get worried and become downhearted.

We have married for five years now. It is not small problem at all. The man’s family disturbs me a lot. Sometimes my husband himself does certain things to me eh because of this problem but I tell him the fault is not from me. He fights me instead of helping me solve the problem. Sometimes even his friends tell him to help me solve the problem but he seems to care less….’menhu nadwen (I don’t know what he is thinking).

**Interviewer:** Alright, it is well. Do you have anything more to say?

Interviewee: No please, but I will try and come again to discuss my challenges with the doctor

Interviewer: That is okay. We would be expecting you. Bye

Interviewee: Thank you. Bye.

**CIN-3**

**Interviewer:** Hello, please good afternoon madam. I am Stephen Mensah Arhin, a PhD student from KNUST, Kumasi. I met you on the first day of your visit to the fertility clinic and discussed with you that we will be checking up on you to find out how the treatment is going and the need for follow up. But unfortunately, we have observed that, you missed some of your appointments with the specialist. For that matter, for that matter, I would like to ask you some few questions as a form of interview to find out why you skipped appointment with the doctor. The interview will be recorded on phone and later transcribed just for the purpose of this study. Please I’m I permitted to do so?

**Interviewee:** yes please, you may go ahead.

**Interviewer:** Thank you. Please what are the reasons why you sometimes skip/discontinue review appointments with clinicians?

**Interviewee:** Hmmm. we will come. There is no issue, but now I can’t really tell whether the problem is from me or from my partner. He does not stay at home. The doctor only gave me treatment and said I should come again when the drug gets finished. He has not been given any drug before except the last time I came. My husband said he will come but he is doing some style. He has also relaxed because he already has two children. Not him alone though. We have two children, except that we are looking for more. So now, we do not know where the fault is coming from. As for me, I have done all the tests the doctor told me to do and he says he cannot identify anything wrong except that he wants to boost my immune system. If my husband was willing to come, I think by now we would have known our fate. Now only God knows when since the power does not lie in my hands alone.

**Interviewer:** thank you very much for these details. Please do you have anything more to say?

Interviewee: Please no. I am only hoping that he would change his mind so we can come for review visit again.

**Interviewer.** Thank you for your time. I am most grateful. Wish you all the best and hoping to see you again. Bye for now.

**Interviewee**: Thank you too for calling. Bye

**CIN-4**

**Interviewer:** Please good afternoon Sir. I am Stephen Mensah Arhin, a PhD student from KNUST, Kumasi. I met you on the first day of your visit to the fertility clinic and discussed with you that we will be checking up on you to find out how the treatment is going. As part of the treatment protocols, you were given some dates to come for review. But unfortunately, you have not honored the appointments with the clinicians as scheduled. For that matter, I would like to ask you some few questions as a form of interview to find out why you skipped appointment with the doctor. The interview will be recorded on phone and later transcribed just for the purpose of this study. No identity is going to be disclosed. Please I’m I permitted to do so? This will take about 10-15 minutes of your time.

**Interviewee:** I see. I am a bit busy now but you may go ahead.

**Interviewer**: Please what makes you discontinue with treatment?

**Interviewee**: It is because of money that is why we stopped coming. We paid a lot of money the last time we came. We did a lot of investigations but doctor said he cannot identify what is wrong with me so we need to do other investigations somewhere, but before the investigation is done, we need to pay Gh 1000 but we did not have the money. That is why we stopped coming although the problem is not solved. We already have problem with finances because my work has spoilt because of Covid-19. My wife too is currently not working. In fact, we have been battling with this issue for the past 5 years since we married and we have spent a lot of money on it. It is not easy bro. We get so worried sometimes. You see, you try to take your mind of it and concentrate on what you are doing. But as you try to focus, your attention is shifted to it from time to time.

**Interviewer: Sorry for that. I understand you perfectly. It shall be well. Just take heart.**

**Interviewee:** Thank you for your concern. I really appreciate. We will try and come when things normalize. At the moment, we do not have enough money that is why you have not seen us for sometime now. We will organize ourselves again and come back later.

**Interviewer:** Thank you too for your time. Hoping to see you soon. Is there anything you may want to share with me again?

**Interviewee:** No please. Thank you

**CIN-5**

**Interviewer:** Please good afternoon Madam. I am Stephen Mensah Arhin, a PhD student from KNUST, Kumasi. I met you on the first day of your visit to the fertility clinic and discussed with you that we will be checking up on you to find out how the treatment is going. As part of the treatment protocols, you were given some dates to come for review. But unfortunately, you have not been coming for review as expected. For that matter, I would like to ask you some few questions as a form of interview to find out why you skipped appointment with the doctor. The interview will be recorded on phone and later transcribed just for the purpose of this study. No identity is going to be disclosed. This will take about 10-15 minutes of your time. Please I’m I permitted?

**Interviewee:** Alright. No problem.

**Interviewer:** Please why did you discontinue with treatment?

**Interviewee:** we have not been coming because the option that they were giving my partner, is like I was not satisfied with it. Is like a sperm count problem, my partner’s own is not low koraa, is zero. So that one, the option that the doctor gave was may be if he wants, they will operate on him and then do what what what what. So I wanted to see if there is any other hospital that I will get another option like given of medications instead of the surgery to improve his situation, ahaaa. You know there are some people a, when they do that and find out, they know that if there is any medicine they can give you a, so that you can take and know what is actually. If they can give option ah, that one, but operation like this, is not something easy. So we were trying to seek advice from others and see if this thing ah, it will be able to solve without passing through that (surgery). That was ehehh my problem but because they didn’t give anything to me or they didn’t say that, I don’t know but that was the only option they gave.

**Interviewer:** did you ask the doctor if there some medicines to manage that instead of surgery?

**Interviewee:** nooo, he was the one who was going to do that but that was the only option he gave my partner. I didn’t say so, but at least if there were some medicine or if he has knowledge about that, ah because we don’t know, have you seen, so if has any idea that, well okay if that is the case ah, try this or that medicine or if it is infection ah, try this medicine and see if it does not work but there was nothing like that. So that is the problem and some small problem I have. If somebody should ask me when you went to that place what happened, I will not know what to say, ahaa so that is the main problem. So, we went to different place and repeated the test, the results was the same but there, they wrote some medicine for him. Under the results that we picked nor, they said oh if you want ah, go try this, you see is good. That one nor, if you don’t have hope ah, you will be having some hope that may be if you are taking ah, everything is always by prayers. So if you think about that ah, may be it has no cure and everything is surgery, it can work a miracle so that was it. So, we went to herbal clinic but we were directed to do the test, especially at a private place. The medicine that they prescribed we wanted to finish with it before we consider going back to the hospital. I have treated ‘white’ (candidiasis) for long time but when we went there, they gave me some treatment claiming I still have white. You know these herbal people, even if you don’t have any problem, they will say you have this and that so take this medicine.

**Interviewer:** Oh, I see. Well, have given me much information. I really appreciate your time. Is there anything you would like to add?

**Interviewee:** For now. That is all I have to say. But you don’t worry, we want to see the outcome of the treatment they gave us so we would know what to do.

**Interviewer:** Thank you for your time. We would be expecting to see again. Have a nice day.

**CIN-6**

**Interviewer:** Please good afternoon. I believe you are doing well. My name is Stephen Mensah Arhin, a PhD student from KNUST, Kumasi. I met you on the first day of your visit to the fertility clinic and discussed with you that we will be checking up on you to find out how the treatment is going. As part of the treatment protocols, you were given some dates to come for review. But unfortunately, we have not been seeing you on your review dates as expected. For that matter, I would like to ask you some few questions as a form of interview to find out why you skipped appointment with the doctor. The interview will be recorded on phone and later transcribed just for the purpose of this study. No identity is going to be disclosed. This will take about 10-15 minutes of your time. Please I’m I permitted to do so?

**Interviewee:** Yes please.

**Interviewer:** Alright. Thank you for permitting me. Please why have you stopped coming for review, which is part of your treatment requirements?

**Interviewee:** I decided to try other alternatives. Actually, I used to come almost every two months for medications but I stopped and went to Accra to see specialist at …. clinic **(name withheld)**. I went there too as well as …. **Clinic (name withheld)**. I later came back for him to see me again later. I wanted to try their system too. This is because I have not got the results as I am expecting. Is like 6 months now since I started treatment at the facility. This is not the first time I am seeking treatment though. Before coming there, I have seen people testifying about the place. That they have got baby. That motivated me to come there too. May be my case is different. Hmmm. I should say because it didn’t take much time for them to get the results they expected. But as for my own, only God knows. I have not given up yet. I know one day one day, it will be my turn

**Interviewer:**  Sorry for that. I know you will definitely get one day. Please I am more grateful for your precious time. Have a blessed day

**CIN-7**

**Interviewer:** Please good evening. I believe you are doing well. My name is Stephen Arhin, a PhD student from KNUST, Kumasi. I met you on the first day of your visit to the fertility clinic and discussed with you that we will be checking up on you to find out how the treatment is going. As part of the treatment protocols, you were given some dates to come for review. But unfortunately, we have not been seeing you on your review dates as expected. For that matter, I would like to ask you some few questions as a form of interview to find out why you discontinued with treatment. The interview will be recorded on phone and later transcribed just for the purpose of this study. No identity is going to be disclosed. This will take about 10-15 minutes of your time. Please I’m I permitted to do so?

**Interviewee:** that is okay. You may go ahead.

**Interviewer:** Thank you for granting me your permission. Please why have not been coming for review visits as ordered by the doctor?

**Interviewee:** My review time happened to be in the lockdown period. More so I have relocated from Cape Coast to Kumasi. So apart from the lockdown, relocation also affected our ability to come for review again. I am continuing treatment in Kumasi at (name withheld) hospital. The doctor told us to repeat all the investigations. We did the investigations and the doctor said everything was okay. He says my husband is okay. My husband was not given any medicine. He says my husband is okay so he is assessing me as well. I was also not given any medicine. The doctor says I should just repeat all the investigations and come back. It is quite expensive doing all the investigations over again. At some point in time, is like we should give up because we were financially drained. But we cannot sit down without getting what we want. We are hoping to get positive outcome. It has not been easy for as at all. Looking at how long we have struggled to conceive but we are not getting it. Sometimes we are demoralized. We even try to seek help from the herbal practitioners sometimes.

**Interviewer:** I see. I can feel your situation. But I think you don’t have to give up, and you may have results one day.

**Interviewee:** Thank you for your concern. We are not given up. Actually, we have not grown so much. We are young couple and we take our consolation from that. Even some people who were in the same situation and older than us have been able to conceive. God is alive.

**Interviewer:** Thank you very much for your time. We wish you all the best and hoping to see you again.

**CIN-8**

**Interviewer:** Please good afternoon. I believe you are doing well. My name is Stephen Mensah Arhin, a PhD student from KNUST, Kumasi. I met you on the first day of your visit to the fertility clinic and discussed with you that we will be checking up on you to find out how the treatment is going. As part of the treatment protocols, you were given some dates to come for review. But unfortunately, we have not been seeing you on your review dates as expected. For that matter, I would like to ask you some few questions as a form of interview to find out why you discontinued with treatment. The interview will be recorded on phone and later transcribed just for the purpose of this study. No identity is going to be disclosed. This will take about 10-15 minutes of your time. Please I’m I permitted?

**Interviewee:** I see. Okay, no problem. You can go on.

**Interviewer:** Thank you for permitting me. Can you please help me know why you stopped coming for review?

**Interviewee:** They keep changing the doctors/specialists that come there. Today this person will come and you change him, tomorrow this person will come and you change him. You call him and he responds ‘I am in Accra’. You call this person and he says ‘ooh I have been transferred’. We were not happy coming there again because erm one person sees your problem and works on it. But the doc who is taking care of us, we call him today I am not there, we call him and he says I am not there. *na ama adeno ay3 basaa, ay3 basaa.*

Is like we have not got anyone who will pass us through the entire process. You see, the thing too is a process. Ahaa is a process, so you start the thing with us and we get to certain stage, then you begin to say mmm I am not here anymore. Then another person comes, then the another person also starts from stage 1. Then we move to stage 2, as we almost get to stage 3, then he also says I am no longer here. Everyone comes, he starts again, and is like the stage 1 that we still maneuver. Emotionally we are down because what we are expecting, we are not getting. That put us off. We are worried as it seems things are delaying. This is not the first time we are seeking care but the situation is the same. Is like we should give up.

**Interviewer:** have you gone to different place since you stopped coming here

Interviewee: *mmm…smh, nhy3da ny3 ayi…smh.* It is (name withheld) that we have been there two times. That place too… “is like try try try. Small children try try”. The students and erh so you see the service is not all that good. You see, is like ucc students who attend to us so you will not get gynecologist who is responsible for such issues to attend to you the way you want. The students will be asking you some things bi a, you see, the things they ask is like noo. Is not all that convincing.

My other challenge is money. I had a little problem at the dispensary. The drugs, the drugs. You see. There is a clear evidence that the prices are always too much at the facility, as compared to when it is purchased outside pharmacies like that of Abura and surrounding communities. The price there is too much. It is something that can put most of the customers off.

**Interviewer:** I see. Sorry for the inconveniences. Do you have anything for us again?

**Interviewee:** For now, no. erm I think those are my concerns.

Interviewer: Thank you very much for your time. Please don’t give up. I hope to see you again. Thank you.

**CIN-9**

**Interviewer:** Please good morning. I believe you are doing well. My name is Stephen Mensah Arhin, a PhD student from KNUST, Kumasi. I met you on the first day of your visit to the fertility clinic and discussed with you that we will be checking up on you to find out how the treatment is going. As part of the treatment protocols, you were given some dates to come for review. But unfortunately, we have not been seeing you on your review dates as expected. For that matter, I would like to ask you some few questions as a form of interview to find out why you discontinued with treatment. The interview will be recorded on phone and later transcribed just for the purpose of this study. No identity is going to be disclosed. This will take about 10-15 minutes of your time. Please I’m I permitted?

**Interviewee:** I hear.

**Interviewer:** Thank you for granting me the permission. Can you please help me know why you have not been coming for review as schedule?

**Interviewee:** hmmm, yes. Is being long time since I came there. Hmm, 3y3 as3m oo (is a problem), is like we have given up. Hmm. We came did all the investigations and there doctor said there is nothing wrong with both of us. Our labs were normal, yet we were not seeing any results so we decided to stop. We don’t know what is actually causing the delay. Is a problem oo. At the moment we are not even at Cape Coast anymore. But we will try and come again. We have moved out of Cape Coast because of work. Is also one of the reasons you have not seen us for some time now. It requires a lot of money if we are to come back there, looking at the distance. That won’t be easy for us at all.

Sometimes we get so worried. My mother usually gets worried too. But we are okay. There is no complain or something like that. Except that I am also not comfortable.

**Interviewer: Alright. It is a very unfortunate experience but the situation will improve. Are you currently planning to continue treatment over there?**

**Interviewee:** Yes. We are planning to see a specialist. The only place we have been told they are also good is a bit far. We need to prepare well financially before we can continue. You know these private places, they charge a lot. Sometimes even if you are going for follow up, you need to pay. Is not easy but we will manage since we have not got what we want.

**Interviewer:** Okay. I understand your situation. I am very grateful for your time. Will call again to check up on you. Have a blessed day.

**CIN-10**

**Interviewer:** Please good afternoon. Please I hope you are good. My name is Stephen Mensah Arhin, a PhD student from KNUST, Kumasi. I met you on the first day of your visit to the fertility clinic and discussed with you that we will be checking up on you to find out how the treatment is going. As part of the treatment protocols, you were given some dates to come for review. But unfortunately, we have not been seeing you on your review dates as expected. For that matter, I would like to ask you some few questions as a form of interview to find out why you discontinued with treatment. Please the interview will be recorded on phone and later transcribed just for the purpose of this study. Your confidentiality is assured. This will take about 10-15 minutes of your time. Please I’m I permitted?

**Interviewee:** Alright. I am okay with it.

**Interviewer:** Thank you. Please why have you not been coming for review visits as expected of you?

**Interviewee:** hmmm. Is about money oo. The treatment cost is not easy. Everything is expensive. Sometimes you feel like coming but if you check yourself, you can see the money is not there. Ahaa. That is why you don’t see me sometimes. More so the man I am staying with, he is doing himself some style bi. He is no more here. He is now in Accra. But I am still in Cape Coast. So I will come. If God permits, I will come. We are not divorced but is being a while that we saw each other. Is like he doesn’t stay at one place. I have been trying that, things will work for me. As for him, he has children. That is it. I am the only person trying hard to get some. Although I have given birth before, he is now sixteen years and I am trying hard to get another one. is not been easy at all. It worries me so much every day. When I keep thinking about the situation, I feel like I should give up and stop worrying myself. Hmmm. But it shall be well.

**Interviewer:** That is quite worrying. But please don’t give up. Try to convince your husband.

**Interviewee:** Hmmm. I have tried aa but I’m tired mpo. But will not give up yet and stick to your advice. Who knows, perhaps I may be lucky for once. Thanks so much

**Interviewer:** Thank you too for your precious time. I will keep in touch with you. Have a blessed day.

**CIN-11**

**Interviewer:** Hello. Good afternoon madam. Please I hope you are doing good. My name is Stephen Mensah Arhin, a PhD student from KNUST, Kumasi. I met you on the first day of your visit to the fertility clinic and discussed with you that we will be checking up on you to find out how the treatment is going. As you were informed on your first visit, you were given some dates to come for review. But unfortunately, we have observed that you have not been following the orders as expected. For that matter, I would like to ask you some few questions as a form of interview to find out why you discontinued with treatment. Please the interview will be recorded on phone and later transcribed just for the purpose of this study. Your identity would not be disclosed. This will take about 10-15 minutes of your time. Please I’m I permitted?

**Interviewee:** No problem. You can go ahead.

**Interviewer:** Thank you. Please why do you skip review visits to the clinic. The doctor was expecting you but you have not been coming for sometime now. What is the cause?

**Interviewee:** I have been coming saa but nothing is coming so I have closed my mind small. When the doctor gives me the drugs, my menses sometimes delay, thinking that I may be pregnant. But when I check for pregnancy, there is nothing there. *Ahaaa.* When the doctor initially told my husband to come and do semen analysis, he said that, his sperms are okay. You know the men…when they are able to impregnate one or two women, they think that, everything about them is correct. He has given birth to a child about one and half month ago with another woman. So me that he is staying with and there is nothing coming, he thinks that is my own problem and I am the cause so I should seek my own help. He didn’t come, although I am still staying with him. Is like he doesn’t care anymore. Even as we are speaking now, he has not come for the tests the doctor requested him to do. That is what I am facing my brother. hmmm. I am worried a lot. I always think about it and emotionally down. How do I keep coming when he is not willing to come too. It puts me down

**Interviewer:** I understand you. This is a cooperate affair. But your case is a bit difficult. So, what is your next line of action?

**Interviewee:** I will come and talk to the doctor to see how best he can help me. So God willing I will come when I am able to put myself together.

**Interviewer:** That is a good idea. I will encourage you to do so. See you next time. Bye.

**CIN-12**

**Interviewer:** Please Good afternoon madam. Please I hope you are doing good. My name is Stephen Mensah Arhin, a PhD student at the department of pharmacology, KNUST, Kumasi. As discussed with you on the first day of your visit to the fertility clinic I am checking up on you to find out how the treatment is going. As you were informed on your first visit, you were given some dates to come for review. But unfortunately, we have observed that you have not been following the orders as expected. For that matter, I would like to ask you some few questions as a form of interview to find out why you discontinued with treatment. Please the interview will be recorded on phone and later transcribed just for the purpose of this study. Your identity would not be disclosed. This will take about 10-15 minutes of your time. Please I’m I permitted?

Interviewee: I am free now so you can go ahead.

**Interviewer:** Alright. Thank you for permitting me. Please why have you decided to discontinue treatment?

**Interviewee:** I have been coming with my partner but nothing has changed. The problem is the same. I was operated last year. My husband has also done his investigations but still the problem persists. Financially too is not being easy. In spite of that, any drug the doctor prescribes, we buy them. Hmmm. The problem is being long overdue….is being long overdue paa. We have been married for over 20 years now. In my first pregnancy, I had ectopic, and since then, none has come again. They did a lot of investigations to ascertain the cause. They said I will be able to conceive again. Later they said is fibroid. I have come for them to remove the fibroid tissue but still, pregnancy is not coming. I am a worried about the condition. I have been asking myself why me. What has gone wrong and what have I done. But only God knows best. We are considering herbal medications too. A friend was testifying about a particular place that they are very good. Initially I did no want to go. But now, hmmm. Is like that is our next option because we want to see results.

**Interviewer:** I see. I understand you and I can see you are doing your best. Don’t give up yet. Is being only three four months now since you started treatment. It is a gradual process so try and come for review. That will give the doctor the opportunity to assess you again and see what is best for you. I am praying you get your baby one day.

**Interviewee:** I here you. I am also certain that my time will come. We need to prepare and come again.

Interviewer: Thank you for your time. Have a wonderful day.

**CIN-13**

**Interviewer:** Please Good afternoon. Please I hope you are doing good. My name is Stephen Mensah Arhin, a PhD from KNUST, Kumasi. As you were informed on your first visit, you were given some dates to come for review. But unfortunately, we have observed that you have not been following the orders as expected. For that matter, I would like to ask you some few questions as a form of interview to find out why you discontinued with treatment. Please the interview will be recorded on phone and later transcribed just for the purpose of this study. Your identity would not be disclosed. This will take about 10-15 minutes of your time. Please I’m I permitted to go ahead and record the interview?

**Interviewee:** Oh no problem. I am free now so you can go ahead.

**Interviewer:** Alright. Thank you for permitting me. Please what make you skip treatment?

Interviewee: Hmmm. My problem has been there for quite some time now. I have spent a lot of money on it. But unfortunately for me, I am just a farmer and do not have too much money. I only come when I have been able to gather some enough money from my farm proceeds. When the money gets finished, I have to suspend my coming until I have been able to raise some money again. As we are speaking now, like you asked me, my time for review has passed but I don’t have the means to come. I am even in the farm right now even though the problem is not solved. Another challenge is the last time we came, it took long time before we could see the doctor. That was very worrying. We almost decided not coming there again because we did not like the experience that day.

**Interviewer:** Sorry for the inconvenience. Try and come again. I don’t this it is like that all the time.

**Interviewee:** Ooh I’m am okay. You know you can’t have everything perfect. We will prepare and come for review very soon.

**Interviewer:** That would be great. You know is not being too long since you started the current treatment. Just comply with treatment and you will have an improved outcome.

**Interviewee:** Thank you. We will surely come again

Interviewer: Thanks for accepting my suggestion in advance. Thanks for your time once again. I believe you will surely come. Have a great day.

**CIN-14**

**Interviewer:** Hello. Please Good morning. Please I hope you are doing good. My name is Stephen Mensah Arhin, a PhD student from the department of Pharmacology, KNUST, Kumasi. I met you on the first day of your visit to the fertility clinic and discussed with you that we will be checking up on you to find out how the treatment is going. As you were informed on your first visit, you were given some dates to come for review. But unfortunately, we have observed that you have not been following the orders as expected. For that matter, I would like to ask you some few questions as a form of interview to find out why you discontinued with treatment. Please the interview will be recorded on phone and later transcribed just for the purpose of this study. Your identity would not be disclosed. This will take about 10-15 minutes of your time. Please I’m I permitted go ahead?

**Interviewee:** Alright, you may go on.

**Interviewer:** Thank you. Please why do you skip review visits to the clinic. What is the cause?

**Interviewee:** My place is very far and I need to come early too. So sometimes when I get to know that I am late, I don’t come at all. This is because when I come and they have finished receiving cards for that day, I have to go back without seeing the doctor. Sometimes I need to come and sleep over in a guest house or hotel but that also requires money. Already, I am paying huge money for some of the tests and medications. This whole thing is very expensive. The last time we came, we spent a lot of money on transportation, investigations and on drugs as well. Sometimes we are worried but because we are the one looking for help, we have reason to complain. We only have to comply except that distance is our enemy.

**Interviewer:** Sorry for that. I know it is not easy financially. And more so National health insurance does not cover these services. What do you plan to do next?

**Interviewee:** Because of the distance, we need to raise some money before we come. Business has not been going well recently.

Interviewer: Alright, we will be expecting you. Thank you for your time

**CIN-15**

**Interviewer:** Hello good evening. Please I hope you are doing good. My name is Stephen Mensah Arhin, a PhD student from KNUST, Kumasi. On your the first day of your visit to the fertility clinic we discussed with you that we will be checking up on you to find out how the treatment is going. As you were informed on your first visit, you were given some dates to come for review. But unfortunately, we have observed that you have not been following the orders as expected. For that matter, I would like to ask you some few questions as a form of interview to find out why you discontinued with treatment. Please the interview will be recorded on phone and later transcribed just for the purpose of this study. Your identity would not be disclosed. This will take about 10-15 minutes of your time. Please I’m I permitted to do so?

**Interviewee:** Alright. You may do so.

**Interviewer:** Thank you for that. Please why is it that for some time now, you have not been coming for review?

**Interviewee:** I came alone the first day. The doctor told me to bring my partner but he is not here. He works in different region and comes here some weekends. You know the hospital does not attend to us on weekends. I have decided to go there so that we can see a doctor at where my husband works. That is the reason why you have not been seeing us as expected. And you know this kind of treatment also goes with money. Ahaa. If we say we want to stay at one place, then it means on of us is going to lose the job we are currently doing. Apart from that, there is no problem at all.

**Interviewer:** That is unfortunate anyway. But so far as you are willing to continue at where your husband is, no problem. If only you can get a clinic over there, that is okay.

**Interviewee:** There is one there but not too close. We need to travel by some distance before we can access the place. All the same, is better than staying apart and not able to come together.

**Interviewer:** Alright. Wishing you the best. Will get in touch with you again

**CIN-16**

**Interviewer:** Hello. Please good morning. Please I believe you are doing well. My name is Stephen Mensah Arhin, a PhD student from KNUST, Kumasi. On your the first day of your visit to the fertility clinic I informed you that we will be checking up on you to find out how the treatment is going. As you were informed on your first visit, you were given some dates to come for review. But unfortunately, we have observed that you have not been following the orders as expected. For that matter, I would like to ask you some few questions as a form of interview to find out why you discontinued with treatment. Please the interview will be recorded on phone and later transcribed just for the purpose of this study. Your identity would not be disclosed. This will take about 10-15 minutes of your time. Please I’m I permitted to do so?

**Interviewee:** Okay.

**Interviewer:** Thank you for allowing me. Please why have you not been coming for review?

**Interviewee:** My husband is always finding excuse to come with me. When I came the first day, the doctor wrote some labs for me to do. He informed me that, my husband has to come for some test. When I went home and I informed him about it, he said he will come but always finding excuses. I can see that, he does not care so much because he has a child already. It seems I am much concerned about the situation than my husband. I am really sad about the situation. i have always been telling him to consider me and come but it seems hmmm. I don’t know what to say. But. You, you will definitely see us on of these days.

**Interviewer:** I hope he cooperates with you so that you could come again for review.

Interviewee: That is my wish. He is a kind of hard person. Sometimes if you talk about it more, he becomes angry. But I am praying he understands me.

Interviewer: It shall be well. Will definitely call you again. Have a blessed day.

**CIN-17**

**Interviewer:** Hello. Please Good morning. Please I hope you are doing good. My name is Stephen Mensah Arhin, a PhD student from the department of Pharmacology, KNUST, Kumasi. On the first day of your visit to the fertility clinic you were informed that, we will be checking up on you to find out how the treatment is going. As you were informed on your first visit, you were told to come for review on certain dates. However, we have observed that you have not been following the orders as expected. For that matter, I would like to ask you some few questions as a form of interview to find out why you discontinued with treatment. Please the interview will be recorded on phone and later transcribed just for the purpose of this study. Your identity would not be disclosed. This will take about 10-15 minutes of your time. Please I’m I permitted go ahead?

**Interviewee:** I see. That’s okay.

**Interviewer:** Thank you. Please why do you skip review visits to the clinic.

**Interviewee:** Hmmm, is a problem oo. Initially I had conviction that, since is a big hospital, I will have better services. But sometimes when you come, you see that the services are not all that good. It did not meet my expectation. Sometimes you need to come as early as possible because of distance but after waiting for a number of hours, then you are told that the doctor will not come. So, you see, you become so disturbed. When you are lucky to have a new person, he may not be experienced as you wish. Yes. So that was my problem. It happened on two occasions that is why I decided not to come again.

**Interviewer:** That was very unfortunate. But don’t give up.

**Interviewee:** Hmmm. We have gone to the herbal people for some medications. We want to see how they can also help us. We have not decided when we are coming to the hospital again. Kindly forgive us. It is not our fault. The system was not favoring us

**Interviewer:** I see. Well, I will call you again to check on you. Thank you very much for your time.

**CIN-18**

**Interviewer:** Please Good evening. Please I hope all is well. My name is Stephen Mensah Arhin, a PhD student from the department of Pharmacology, KNUST, Kumasi. On the first day of your visit to the fertility clinic you were informed that, we will be checking up on you to find out how the treatment is going. As you were informed on your first visit, you were told to come for review on certain dates. However, we have observed that you have not been following the orders as expected. For that matter, I would like to ask you some few questions as a form of interview to find out why you discontinued with treatment. Please the interview will be recorded on phone and later transcribed just for the purpose of this study. Your identity would not be disclosed. This will take about 10-15 minutes of your time. Please I’m I permitted go ahead?

**Interviewee:** well, I remember you now. Hope you are doing well too?

**Interviewer:** I am also good. Thank you. Please why have you not been coming for review as ordered by the doctor.

**Interviewee:** My major challenge has been finances. We need to pay for services anytime we come. Besides, we come from a very far place. We come all the way from Praso so is not easy to follow all the orders they give us. That is not our wish though, but if for any reason, we delay a bit, then we have to suspend our coming because we may not meet the doctor when we get there late. Coming with my partner from such a distance requires a lot of money. Sometimes, when the medications are writing for us, we are unable to buy all. That is our main reason for not coming

**Interviewer:** I understand you. Treatment is not all that cheap. So, what is the way forward now?

**Interviewee:** We will come but we can’t tell when. When we are able to mobilize some more money, we will come again. We wish we had gotten the results we are looking for but it seems our financial strength is not helping us. God is in control

**Interviewer:** Alright. We would be expecting you. Have a wonderful day. Thanks for your precious time.

**CIN-19**

**Interviewer:** Please Good morning. Please I hope you are good. My name is Stephen Mensah Arhin, a PhD student from KNUST, Kumasi. On the first day of your visit to the fertility clinic you were informed that, we will be checking up on you to find out how the treatment is going. As you were informed on your first visit, you were told to come for review on certain dates. However, we have observed that you have not been following the orders as expected. For that matter, I would like to ask you some few questions as a form of interview to find out why you discontinued with treatment. Please the interview will be recorded on phone and later transcribed just for the purpose of this study. Your identity would not be disclosed. This will take about 10-15 minutes of your time. Please I’m I permitted go ahead?

**Interviewee:** Alright. You may go on

**Interviewer:** Thank you. Please why have you not been coming for review as ordered by the doctor.

**Interviewee:** We stopped coming because we were not seeing any results. We were so disturbed about the delayed outcome so we decided to seek solution elsewhere. Now, although the problem is not solved, we are hoping for the best. So we tried different place too. We are still taking our medications.

**Interviewer**: I see. Per your records, you started the treatment just about 13 weeks ago. Don’t you think is too early to quit?

**Interviewee:** Hmmm. Is not easy, there is too much pressure on us. We need to get the problem solved as early as possible.

**Interviewer**: That is a kind of hasty decision though. So, are you planning to come back?

**Interviewee:** we can’t really tell. It will depend on the outcome of the treatment now

**Interviewer:** Alright. I am most grateful for your time. Will get in touch with you again

**CIN-20**

**Interviewer:** Please Good afternoon. Please I hope all is well. My name is Stephen Mensah Arhin, a PhD student from the department of Pharmacology, KNUST, Kumasi. We met you on the first day of your visit to the fertility clinic and informed you that we will be checking up on you to find out how the treatment is going. As you were informed on your first visit, you were told to come for review on certain dates. But, we have observed that you have not been following the orders as expected. For that matter, I would like to ask you some few questions as a form of interview to find out why you discontinued with treatment. Please the interview will be recorded on phone and later transcribed just for the purpose of this study. Your identity would not be disclosed. This will take about 10-15 minutes of your time. Please I’m I permitted go ahead?

**Interviewee:** That is okay.

**Interviewer:** Please why have you not been coming for review visits as expected of you?

**Interviewee:** Hmmm. My husband is doing himself some way. As if he is not interested in the marriage anymore because of the problem. He shows less concern about seeking care. Now it seems I am giving up. Yes. Is a big challenge but what can I do? For sometime now, he does not even stay at home. Then when you talk about this issue, he becomes annoyed sometimes. I am even stranded now because of his behavior. As for my marriage, it is on the verge of collapsing. I see that. That is why my husband does not care anymore. He is seeing some other ladies. I am very worried. Life seem meaningless sometimes.

**Interviewer:** I can feel your situation. it is not easy. But just take heart and don’t fight him. He may change.

**Interviewee**: That is my wish anyway. I am always praying to God to change him. I will come again whenever he is ready.

Interviewer: It would be nice to see you again. Thank you very much for your time.

# **Clinicians**

HP-1

**Interviewer:** Good morning doctor. I believe you are doing well. I am Stephen Mensah Arhin, a PhD student from KNUST. Good to meet you again. Please as you are already aware, as part of my studies, I am conducting interviews on why infertile patients Discontinue their treatment prior to achieving the desired results. Since you are a key stakeholder in the care for fertility patients, I would like to seek your opinion on why you think the patients drop out from treatment. Please the interview will be recorded and transcribed later just for the purpose of this study. Your confidentiality is fully assured. Please I’m I permitted to go ahead? This interview will last for about 10-15 minutes.

**Interviewee:** Alright. That is okay.

**Interviewer: Thank you. Please how long have you been managing fertility patients?**

**Interviewee:** I have been treating infertile patients for the past 12 years. I have worked in different facilities including both government and the private sector.

**Interviewer:** That is quite some time now. It means you have the experience. Please can you tell me some of the reasons that make infertile patients discontinue pharmacotherapy prior to achieving conception.

**Interviewee:** As for that one, is mostly because of money issues. Lack of funds to pursue the investigations and treatment. Second one is unwillingness of the husbands to come and do investigation. Like you ask them to come for semen test and they don’t show up. Ahaa. These are the two main reasons. Thus, lack of funds and male partners not willing to come for evaluation, especially semen analysis. So, the lack of support from partners is a major contributing factor. Most of the men see themselves as capable. The perception that women are the ones to give girth causes most of them men to behave in this way. They think only the women need treatment.

**Interviewer:** I see. That is interesting. So, when it happens in this way, what do you do?

**Interviewee:** mostly the women come back with these complains after they have been told to bring their partners. Some end up even crying because their husbands are not cooperating. We try to assure them. Ahaa. We encourage them not to give up. Sometimes we tell them to discuss with an elderly person or their pastor who can convince the man to come. Yes. Is a whole lot

Interviewer: I see. The work is not easy. Thank you so much for your time with me. I am most grateful.

**HP-2**

**Interviewer:** Good afternoon nurse. I believe you are doing well. I am Stephen Mensah Arhin, a PhD student from KNUST. Please as you are already aware, as part of my studies, I am conducting interviews on why infertile patients discontinue their drug therapy prior to achieving the desired results. As one of the health professionals who provides direct fertility patients. I would like to seek your opinion on why you think the patients drop out from treatment. Please the interview will be recorded and transcribed later just for the purpose of this study. Your confidentiality is fully assured. This interview will last for about 10-15 minutes. Please I’m I permitted to go ahead?

**Interviewee:** Okay Sir.

**Interviewer:** Thank you. Please how long have you been working at the fertility clinic?

**Interviewee:** I have been attending to fertility patients for the past 11 years.

**Interviewer:** Alright. Please why do you think infertile patients discontinue pharmacotherapy prior to achieving conception?

**Interviewee:** One of the main reasons I think clients don’t come is because of distance. You know, some of them come all the way from Takoradi and other far places. That is one of the hinderances for them. Another thing could also be the client’s personal reasons. That is the slow rate at which things work for them so they change facility. They try to seek treatment from many places. You see. To some of them, the society expects them to give birth right after marriage. Especially the young couples. That is what I have noticed. Sometimes you take their contact and call them. When you ask why you have not seen them for long, and whether they have been able to conceive. They will you so many reasons. My money has finished, the fare is too high, and I am considering some other place. Yes. The reasons are many. That is what I can tell you. For that matter, some decide to come at their own comfort time.

**Interviewer:** I see. Very interesting. You have really given me much information. I really appreciate. Do you have any more to say?

**Interviewee:** Sometimes is difficult to even monitor whether the treatment is effective or not. But that is their challenge so what can we do. Since we can’t solve it for them, we just remain quiet.

**Interviewer:** Alright. thank you for your precious time. I am very grateful.

**HP-3**

**Interviewer:** Good afternoon doctor. I believe you are doing well. I am Stephen Mensah Arhin, a PhD student from KNUST. Please as part of my research, I am conducting interviews on why infertile patients discontinue drug therapy prior to achieving the desired results. As a fertility specialist who provides direct care to the patients. I would like to know from your side, why you think the patients drop out of treatment. Please the interview will be recorded and transcribed later just for the purpose of this study. Your identity is not going to be disclosed. This interview will last for about 10-15 minutes. Do I have your permission to go ahead?

Interviewee: Of course, yes. You are free to start.

**Interviewer:** Thank you doctor. Please can you tell me how long you have treated fertility patients, and why you think some patients drop out of treatment while they are yet to achieve their desired outcome?

**Interviewee:** I have been in the job for the past 19 years. I have worked in three different hospitals in general. In all these years, what I have observed as the main reason for drop out is because they are fed up. Since they don’t see better result after coming for some time, they tend to give up. Usually, within few months of therapy, they are expecting to conceive. For that matter, most of them move about one place to the other just to get instant results. Sometimes too, they decide to change facility just to experiment other places. This happens when they thing the facility is not helping them achieve their aim. Another could also be due to the charges involved. You know they have to pay huge money for investigations and treatment. And the cost of treatment is also expensive. Yes. Another reason is also that, some of them, their male partners do not come for evaluation. They leave the women alone. When they women try their best to convince the men and they still refuse to come, they tend to give up. So, these are the important reasons why I think they are not coming.

**Interviewer:** Very well said. These are very important revelations. So, what do you do if he men don’t come? Do you go ahead to treat the woman or you ignore them?

**Interviewee:** You see. When the women come alone and you see they way they are desperate, you have to treat them after they have gone through the necessary investigations. Some of them, their labs may all be normal but we still go ahead to write some treatment for them. Some even go ahead to ask for treatment for their partners, but hmmm is like they don’t understand when you tell them that is not the way to go.

**Interviewer:** I see. I have been schooled today. Thank you so much for such an important information. I am really grateful. Have a blessed day.

**HP-4**

**Interviewer:** Good afternoon nurse. I believe you are good. I am Stephen Mensah Arhin, a PhD student from KNUST. Although we have met before, I still need to introduce myself anyway. Please as part of my research, I am conducting interviews on why infertile patients discontinue drug therapy prior to achieving the desired results. As a nurse who has provided direct care to fertility patients. I would like to know from your side, why you think the patients drop out of treatment even before they achieve their desired outcome. Please the interview will be recorded and transcribed later just for the purpose of this study. Your identity is not going to be disclosed. This interview will last for about 10-15 minutes. Do I have your permission to go ahead?

**Interviewee:** Okay.

**Interviewer:** Thank you Maame Nurse. Please can you tell me how long you have been taking care of fertility patients

**Interviewee:** I have worked as a nurse at the fertility clinic for the past 13 years. Not in this facility alone though.

Interviewer: Please can you tell me why you think some patients drop out of treatment prior to achieving pregnancy?

**Interviewee:** Well, what I have observed is that most of the patients have financial challenges. The investigations and treatment are very expensive. This tend to put many of them off after they have started the whole process. Another reason is also lack of cooperation from male partners. They fail to come along with the women. The doctors are supposed to assess them together so if they don’t show up, it demoralizes the women. This also contributes to their default. You know, these services are not covered by health insurance so the clients have to bear the cost themselves. Again, I think some of them too faces the problem of stigmatization. Some even play hide and seek with their neighbors when coming for treatment. Is like when people see them, they look at them in some way. Yes. This forces some of them to seek treatment at places far away from their communities. Some even travel miles to access treatment.

**Interviewer:** That is very insightful. I have really enjoyed the conversation. Do you have any more to say?

**Interviewee:** Well, that is all I have to say for now. But in case you need more information regarding your study, I am ever ready to assist if is within my jurisdiction.

Interviewer: Thank you for your time. I really appreciate. Have a wonderful day.

**HP-5**

**Interviewer:** Good evening doctor. I believe you are good. I am Stephen Mensah Arhin, a PhD student from KNUST. Nice to meet you again. Please as part of my research, I am conducting interview on why infertile patients discontinue drug therapy prior to achieving the desired results. As a doctor who has provided direct care to fertility patients. I would like to know from your side, why you think the patients drop out of treatment prior to achieving conception. Please the interview will be recorded and transcribed later just for the purpose of this study. Your identity is not going to be disclosed. This interview will last for about 10-15 minutes. Do I have your permission to go ahead?

**Interviewee:** Alright. I am ready now.

**Interviewer:** Thank you Doctor. Please can you tell me how long you have managed infertility patients?

**Interviewee:** That is over ten years now. At least we have got some experience in the profession. Hahaha. Well I’m ready so go ahead

Interviewer: Than you doc. Please my question is a kind of straight forward one. Can you tell me why you think some patients drop out of treatment prior to achieving conception?

**Interviewee:** What I can say is that, most of them don’t always feel comfortable coming for treatment. They don’t want some of the people close to them know that, this is what they are suffering from. They think that others will be gossiping about them. This forces some of them to seek care at places far from where they live or work, as you might be aware, some of them already feel the pressure of social stigma. Others too have financial challenges. They always complain of expensive investigations and cost of medications. That is why most of them discontinue with treatment. For some couples, the men fail to avail themselves for investigation. The women are mostly left stranded with the men busily going about their normal duties. Like you ask the women. Where is your husband? And they will then tell you he has gone to work or this or that. That they are not ready to do the investigations. It goes on and on and on.

**Interviewer:** I see. thank you for such information. As we are bringing the conversation to an end. Is there anything more you would like us to know?

**Interviewee:** Those are the reasons I think confronting them for now. We will keep doing our best to help them. You know this whole condition comes with both physical and psychological problems. The dilemma they go through is sometimes unbearable. This forces some of them to even take hasty decisions by discontinuing treatment because are not getting what they want.

**Interviewer:** Thank you so much for lending me some of your precious time. Have a blessed day.

**HP-6**

**Interviewer:** Good morning Nurse. I believe you are good. I am Stephen Mensah Arhin, a PhD student from KNUST. Please as part of my research, I am conducting interviews on why infertile patients discontinue drug therapy prior to achieving the desired results. As a nurse who provides direct care to fertility patients. I would like to seek your opinion on why you think the patients drop out of treatment prior to achieving conception. Please the interview will be recorded and transcribed later just for the purpose of this study. Your identity is not going to be disclosed. This interview will last for about 10-15 minutes. Do I have your permission to go ahead?

**Interviewee:** No problem my brother. At the moment, I am less busy so you can go ahead

**Interviewer:** Thank you. Please can you tell me how long you have cared for fertility patients?

**Interviewee:** That is over 8 years now.

**Interviewer:** Thank you. Please can you tell me why you think some patients drop out of treatment prior to achieving conception?

**Interviewee:** Thank you my brother. I think the main problem is the men. That is what I have observed over the years. They give the women difficult time. Is like the burden of the women alone. May be that is how our society has made is look like. Some of the men don’t really support the women. They refuse to come for the investigations. That is why most of them are not able to continue with treatment. Some of them too, when they come and they don’t achieve the results as expected, they give up. That is it. That is what I have observed so far. I always try to encourage them to focus on the treatment but not to give attention to people around because if you do that, you can focus on treatment. Everybody has their own problem but they don’t come and tell anybody. So why should you let others intimidate you. That is me.

**Interviewer:** Wow. Really interesting. So, going forward, what do you think will be the solution to some of these problems?

**Interviewee:** I think the men should change their mentality. Infertility is not one person’s business. So, they stop putting all the burden on the women alone

**Interviewer:** Thank you so much for your time. I really appreciate. Have a nice day.

**HP-7**

**Interviewer:** Good afternoon in charge. Please I believe you are doing well. I am Stephen Mensah Arhin, a PhD student from KNUST. Is being a while ago. Please as part of my research, I am conducting interviews on why infertile patients discontinue drug therapy prior to achieving the desired results. As a nurse who provides direct care to fertility patients. I would like to seek your opinion on why you think the patients drop out of treatment prior to achieving conception. Please the interview will be recorded and transcribed later just for the purpose of this study. Your identity is not going to be disclosed. This interview will last for about 10-15 minutes. Can I go ahead please?

**Interviewee:** Yes, I am ready.

**Interviewer:** Alright. Thank you. Please can you tell me how long you have provided care to fertility patients?

**Interviewee:** That is over 9 years now.

Interviewer: I see. Please can you tell me why you think some fertility patients discontinue treatment prior to achieving conception?

**Interviewee:** I can’t really say much since most of their reasons are personal. However, I think distance might be a contributing factor. Some do come from far places. You know our clinics operates once in a week, so if by any reason anything should come across them, they are not able to come as expected. Others also say that they are occupied by work. Especially the men. When they are to come for review, they find excuses with their work. Most of the women seem to bear the responsibility alone. Work is one of the main reasons they skip reviews. Some say without working to raise money, they cannot cater for the cost of treatment. They are sometimes confronted with the reality of high cost of treatment. As such, they are not able to come for review as expected of them.

**Interviewer:** Of course, distance can really hit them hard, especially those who come from far places. Please do you have anything to say again.

**Interviewee:** No please.

**Interviewer:** thank you for your time. God bless you.

**HP-8**

**Interviewer:** Good afternoon doctor. I believe you are doing great. I am Stephen Mensah Arhin, a PhD student from KNUST. Please as part of my research, as you are already aware with most of the patients not showing up for review visits, I am conducting interviews on why infertile patients discontinue drug therapy prior to achieving the desired results. As a doctor who has provided direct care to fertility patients. I would like to know from your side, why you think the patients drop out of treatment prior to achieving conception. Please the interview will be recorded and transcribed later just for the purpose of this study. Your identity is not going to be disclosed. This interview will last for about 10-15 minutes. Do I have your permission to go ahead?

**Interviewee:** Yes. I am okay with it.

**Interviewer:** Thank you Doctor. Please can you tell me how long you have managed infertility patients?

**Interviewee:** That is over 15 years now.

**Interviewer:**  Than you doc. Please doctor, can you tell me why you think some patients drop out of treatment prior to achieving conception?

**Interviewee:** What I can say is that, most of them want instant results. But is a process so they easily give up. Most of them do not have the patients to wait for long. To some of them, the mere fact that they have come for treatment and within some few days they are not seeing results as expected, they tend to give up. Another reason is the cost of treatment. You know, national health insurance does not cover the services so the clients bear all the cost. Is something that can put them off. The third reason is distance. No access to it where they live, especially those in rural areas. They have to travel all the way to the city to access these services since is not available over there. That is the third reason. The fourth reason is that, they feel stigmatized. They feel people will be asking them why they are always going to hospital. The doctors they are used to, they don’t come to meet them all the time. Me, myself, my days are only Tuesdays so when sometimes they come and I am not around, they tend to go without coming back. So, these are the four main reasons.

Some of them become psychologically down when they don’t get the results they are looking for on time. For that matter, they tend to quit and seek treatment elsewhere. Most of them do not have the patience to wait for long. Some of these things happen because of societal pressure. To some of them, the mere fact that they have come for treatment and within some few days they are not seeing results as expected, they tend to give up.

**Interviewer:** Thanks so much for such a detailed update. You have actually furnished me with a lot of information. Please anything to add again?

**Interviewee:** No. That’s it for now. That is what I have observed over the years

**Interviewer:** Thank you for your time. I am most grateful. Have a great day
