# Supplementary material for: Pharmacotherapy of infertility in Ghana: Why do infertile patients discontinue their fertility treatment?
Source: PLoS One. 2022 Oct 17;17(10):e0274635. doi: 10.1371/journal.pone.0274635 (PMC9576038; doi:10.1371/journal.pone.0274635)
Supplement: Attachment — Submitted filename: Response to reviewers 1.docx [file pone.0274635.s002.docx]

**Editor’s comments**

Thank you for submitting your manuscript to PLOS ONE. After careful consideration, we feel that it has merit but does not fully meet PLOS ONE’s publication criteria as it currently stands. Therefore, we invite you to submit a revised version of the manuscript that addresses the points raised during the review process.

In addition to comments provided by the two reviewers, the please consider the following:

**Comment**

Line 26: Consider revision to "The study employed an exploratory qualitative design"

**Response**

The change has been effected in the revised manuscript. (line 26, pg 2).

**Comment:**

Line 26-27: It is enough to say "purposive sampling technique"

**Response**

The change has been effected in the new manuscript (line 26, pg 2).

**Comment**

Line 28: Write ‘8’ in word

**Response:**

The number ‘8’ has been written in full in the new manuscript (line 28, pg 2)

**Comment:**

Line 29: Consider changing "were sampled and interviewed" to just "were interviewed"

**Response:**

The change has been effected in the revised manuscript (line 29, pg 2).

**Comment:**

In the abstract, state the data analysis approach in a short sentence.

**Response:**

The data analysis approach has been mentioned in the abstract in the revised manuscript (line 31).

**Comment**

Line 32: Consider revising to: “The major reasons for discontinuation of infertility medical services included….”

**Response**

The change has been effected in the revised manuscript (line 32).

**Comment**

Line 37-39: It might be better to say the insight provided rather that say it will provide insight. The current statement sounds like a justification for the study, not a recommendation from the findings, as might be the intention of the authors.

**Response**

The change has been effected in the revised manuscript (line 37-38).

**Comment**

Line 94: This initial statement does not make your study population clear. Twenty couples ordinarily means 20 pairs of persons, making 40 persons.

**Response**

The term ‘couples’ has been changed to ‘patients’ in the revised manuscript.

**Comment:**

Also, please indicate that you did not interview both members of each couple pair, and why not.

**Response:**

None of the 20 respondents were from the same couple pair. The telephone calls were made to the number(s) provided by the couple-pair during their first visit. However, any of the partners who was available and willing to participate in the study was recruited.

**Comment**

Please ensure to remove any personal identifying information from your manuscript, including names of organizations where identifying them is not essential to the information being provided. You must justify the inclusion of names that identify any entity.

**Response**

Names of organizations that are not essential to the information being provided have been removed from the revised manuscript.

**Comment**

Concerning your suggestions on the approach to data collection, infertility is a sensitive issue and FGD may still not be the most appropriate fora for data collection, particularly when the proposed participants are currently experiencing infertility.

**Response**

Thank you for your comment regarding the approach to data collection we suggested as recommendation for further research. The suggestion of FGD has been removed from the revised manuscript.

**Comment**

In line with the comments of one of the reviewers, consider removing the word "subfertile" from your title.

**Response**

The term “subfertile” has been removed from the title of the revised manuscript.

**Comment**

Do pay attention to typographical and grammatical errors.

**Response**

The authors are grateful to the Editor for his constructive comments and suggestions in reshaping out manuscript.

The revised manuscript has been proofread by people and typographical and grammatical errors.

**RESPONSE TO REVIEWER 1**

**Comment:**

In the title you talk about choices that subfertile couples make, but I am not sure how representative the term couples is of the study population?

**Response:**

It is true that the female respondents were more than the male respondents. Throughout the study, it was observed that the males were less forthcoming when it comes to cooperating with treatment and willingness to participate in the study. This has largely been attributed to the cultural setting in African, which places the responsibility of childbirth on the doorsteps of women. This has been well-elaborated in the manuscript. As such, we have revised the term “couples” to “patients” in the title.

**Comment:**

Some small changes which would make some things clearer for me:

line 72, pg 4 - change to 'for infertility' instead of 'of infertility'

**Response:**

The change has been effected (line 74, pg 4).

**Comment:**

line 73, pg 4 - add Metropolis to Cape Coast, as you later refer to just 'the metropolis' this confused me, and then you later call it Cape Coast Metropolis

**Response:**

The change has been effected (line 82, page 4).

**Comment:**

line 76, pg 4 - to enable stakeholders 'to' develop (word missing)

**Response:**

The change has been effected (line 78, page 4).

**Comment:**

lines 94-96, pg 5 - I would like to see this more clearly, on first reading it appears you interviewed 20 couples, but you then say you interviewed 14 women and 6 men. Maybe be specific that you interviewed 20 clients, which were a combination of 14 women and 6 men - but were any of these couples?

**Response:**

The suggestion has been well noted and correction effected in the revised manuscript. Please refer to lines 96-97 on page 5.

On the question regarding whether any of the respondents were couples, the telephone calls were made to the number(s) provided by the couples during their first visit. However, any of the partners who was willing to participate in the study was recruited. As such, none of the 20 respondents were from the same couple pair.

**Comment:**

line 130 - pg 7 - who did the transcription? and also did you use a specific type of thematic analysis?

**Response**

Two of the authors inductively coded four of the transcripts (two from CIN and two from HP) independently. The initial codes were review6d by three of the authors for consistency before a consensus was reached on the codes and the coding system. The first author then coded all the transcripts using the coding system agreed upon (line 145-149, pg 8).

On the question of a specific type of thematic analysis, a thematic content analysis was used to analyze the data (line 143-144, pg 8).

**Comment:**

line 132, pg 7 - reword 'two of the authors coded few' this reads as though they coded a minimal amount - might be useful to say how many and which (HCP or clients).

**Response:**

Two of the authors inductively coded four of the transcripts (two from patients and two from the healthcare personnel). As such the changes have been effected in the revised manuscript. (lines 146-147).

**Comment:**

line 135, pg 7 - It feels more accurate to say that themes were identified than that they emerged.

**Response:**

We have changed the term “emerged” to “identified” in the revised manuscript (line 151, pg 8).

**Comment:**

line 138, pg 8 - I think you mean that you were maintaining confidentiality, the opposite to disclosing the identity of the participants?

**Response:**

The correction has been effected in the revised manuscript. (Please refer to page 8, line 134).

**Comment:**

line 190, pg 10 - 'live' birth (also line 430)

**Response:**

The correction has been effected. (line 212, page 12).

**Comment:**

line 433, pg 21 - you talk about internal and external pressures - which do you mean specifically?

**Response:**

The sentence has been clarified in the revised manuscript (line 457-458, pg 23).

**Comment:**

line 485, pg 24 - midst, not mist

**Response:**

The word has been spelt correctly in the revised manuscript. Line 518, page 27.

**Comment**

I really enjoyed reading this, thank you. And I think you have made some really important points, particularly about the abdication of responsibility of the male partners.

There has been much made of the 'delay' to treatment outcomes - do you have a sense, or data to indicate what sort of time frames that are contributing to the drop out of treatment?

I think that this article may need some editing for language and clarity, but the data support the conclusions and suggestions you have made for how services could be improved.

**Response**

Authors are grateful to the reviewer for her kind comments about our manuscript. We also thank her for the constructive comments which have made the revised manuscript better.

From the observations made in the study as well as responses from health professionals, a delay in expected outcome for about 1–3 months for males and 2-4 months for the females, usually caused them to abdicate treatment. We have included the time frames in the revised manuscript.

The manuscript has been proofread by people with English as their first language.

**Reviewer #2: Review PONE-D-22-12329**

This a very important qualitative interview study on why couples in Ghana discontinue fertility treatment (pharmacotherapy). A topic where we have very limited knowledge from low/middle income countries. Globally, infertility is one of the most frequent diseases among reproductive-aged women and men. In middle and low-income countries the access to fertility treatment is limited and is not affordable for many couples with infertility. The study is carefully conducted, data clearly presented and the manuscript is easy to follow.

I have a number of minor suggestions.

**Terminology**

I suggest to follow the terminology in the International Glossary on Infertility and Fertility Care, 2017 (Zegers-Hochschild et al., Human Reproduction, 2017). My suggestion is to use “infertility” as this is clearly defined (and not “subfertility” that is usually not well-defined); fertility care, fertility clinics, fertility treatment and in line with this fertility patients.

Throughout the manuscript is used both “clients” and “fertility patients”. I suggest to use only one term (fertility patients). Similarly, is used both “infertility” and “subfertility”. I suggest infertility as stated above.

**Response:**

We thank the reviewer for drawing our attention to this. We have replaced “subfertility” with “infertility” in the revised patients.

**INTRODUCTION**

**Comment**

Is well-written and provides a good overview. I suggest to include a few more papers on infertility in Sub-Saharan countries. Search e.g., for papers in Pubmed published by Silke Dyer, Johanne Sundby, Florence Naab and others and you will find papers e.g., on access to fertility care, the psychosocial impact of infertility, studies on the impact of male infertility, how fertility patients assess treatment. Some could probably also be included in the Discussion section.

**Response**

The recommended papers have been read as suggested. The introduction and the discussion sections have been updated accordingly.

**MATERIALS AND METHODS**

**Comment**

I suggest to add either a table or a short overall description of the study participants (besides their gender). For the fertility patients – e.g., age range, previous reproductive history (have child/children or not), previous treatment. For the health care professionals – e.g, number of years in fertility care. This info should be provided on a group level, so the study participants could not be identified.

**Response**

Tables indicating the overall description of study participants have been provided in the revised manuscript.

**Comment**

I suggest to put the paragraph on Ethical approval as the last section before Results.

**Response**

The changes have been effected in the revised manuscript

**Comment**

Please, provide a reference to the thematic analysis used.

**Response**

Reference has been provided in the revised manuscript.

**Comment**

The description of how you assigned codes to the interview transcripts is not a Result and should be placed in the M&M section.

**Response**

The changes have been effected as suggested.

**RESULTS**

I suggest you provide a table showing themes from interviews with fertility patients and with health care professionals, respectively. To help the reader to have an overview.

Otherwise the result section is easy to follow and quotes selected are illustrative for the theme.

**Comment**

In quotes, I would recommend not to mention e.g., name of fertility clinics in order not to reveal study participant identity.

**Response**

Names of fertility clinics have been removed from the revised manuscript.

**Comment**

I would prefer Psychological distress (p. 15 , line 299) instead of disturbances (which sounds closer to psychiatric disorders).

**Response**

The correction has been effected in the revised manuscript (line 323, page 18).

**Comment**

Discussion is relevant. I found it interesting that the fertility patients’ do not mentioning stigma as a reason for discontinuation, but it was mentioned by the health care professionals. Could this difference be discussed in short?

**Response**

The discussion on sigma has been updated in the revised manuscript (line 501-509, page 26).
