# Supplementary material for: Pharmacotherapy of infertility in Ghana: Why do infertile patients discontinue their fertility treatment?
Source: PLoS One. 2022 Oct 17;17(10):e0274635. doi: 10.1371/journal.pone.0274635 (PMC9576038; doi:10.1371/journal.pone.0274635)
Supplement: Attachment — Submitted filename: RESPONSE TO THE EDITOR AND REVIEWERS COMMENTS.docx [file pone.0274635.s003.docx]

**Response to editor’s and reviewers’ comments**

We are very much grateful to the editor and the reviewers for their meaning contributions towards reshaping of our manuscript.

**Response to Editor’s comment**

**Comment:** We noticed that your study included 20 participants, however the "occupation" row in Table 1 adds up to 21 individuals. If it was possible to provide multiple answers to this question, please indicate this. Otherwise, please ensure to correct the "occupation" row with the correct value.

**Response:** This was an error, and correction has been effected in the revised manuscript.

**Comment:** We noticed that in Table 1 there is a difference in the capitalization of "Male" vs "female"

**Response:** Appropriate changes have been made to ensure consistency in the revised manuscript.

**Comment:** Please check your other tables for similar inconsistencies (numbers and grammar).

**Response:** Other tables have been checked and necessary changes effected in the revised manuscript.

**Response to reviewer 2 comments**

**Comment:** Most places the author states "infertile patients" and fewer places "fertility patients" (e.g., Table 3, and in sub-heading line 205). I suggest to be consistent throughout the manuscript and use one term only. As previously mentioned, I prefer "fertility patients"; however, this is not a requirement.

**Response:** We are grateful to you for drawing our attention to this error. The necessary corrections have been effected to ensure uniformity and consistency. However, the term ‘infertile patients’ has been maintained in the revised manuscript.

**Comment:**  I would avoid the term "drop-out" of fertility treatment, as "drop-out" could indicate something that just happened passively. Studies - including your own - clearly indicates that fertility patients who discontinue treatment has been actively through a decision-making process.
On p. 6, Sampling, I suggest e.g. to revise to "20 fertility patients who had discontinued treatment " and in Table 1 to change "drop-out time" to Treatment discontinuation. I am not sure whether it is discontinuation after 1-3 months of being in fertility treatment?

**Response:**  The suggested corrections have been effected accordingly in the revised manuscript.
On the issue of discontinuation, the 1-3 months means the average duration after which the patients discontinued their treatment.
